# Stabilization and Augmentation of Circulating AIM in Mice by Synthesized IgM-Fc

**DOI:** 10.1371/journal.pone.0097037

**Published:** 2014-05-07

**Authors:** Toshihiro Kai, Tomoko Yamazaki, Satoko Arai, Toru Miyazaki

**Affiliations:** 1 Laboratory of Molecular Biomedicine for Pathogenesis, Center for Disease Biology and Integrative Medicine, Faculty of Medicine, The University of Tokyo, Tokyo, Japan; 2 CREST, Japan Science and Technology Agency, Tokyo, Japan; State University of Rio de Janeiro, Biomedical Center, Institute of Biology, Brazil

## Abstract

Owing to rapid and drastic changes in lifestyle and eating habits in modern society, obesity and obesity-associated diseases are among the most important public health problems. Hence, the development of therapeutic approaches to regulate obesity is strongly desired. In view of previous work showing that apoptosis inhibitor of macrophage (AIM) blocks lipid storage in adipocytes, thereby preventing obesity caused by a high-fat diet, we here explored a strategy to augment circulating AIM levels. We synthesized the Fc portion of the soluble human immunoglobulin (Ig)M heavy chain and found that it formed a pentamer containing IgJ as natural IgM does, and effectively associated with AIM *in vitro*. When we injected the synthesized Fc intravenously into mice lacking circulating IgM, it associated with endogenous mouse AIM, protecting AIM from renal excretion and preserving the circulating AIM levels. As the synthesized Fc lacked the antigen-recognizing variable region, it provoked no undesired immune response. In addition, a challenge with the Fc-human AIM complex in wild-type mice, which exhibited normal levels of circulating IgM and AIM, successfully maintained the levels of the human AIM in mouse blood. We also observed that the human AIM was effectively incorporated into adipocytes in visceral fat tissue, suggesting its functionality against obesity. Thus, our findings reveal potent strategies to safely increase AIM levels, which could form the basis for developing novel therapies for obesity.

## Introduction

The proportion of the population suffering from obesity has been rapidly increasing because of drastic changes in lifestyle and eating habits in modern society. Obesity has become one of the most important public health problems, as it induces metabolic syndrome, which is now widespread and more deadly than first thought. Enlargement of adipocytes in visceral fat tissue is accompanied by recruitment of proinflammatory macrophages in fat, causing chronic, low-grade inflammation locally and systemically [1]–[3]. This subclinical state of inflammation induces insulin resistance which leads to a variety of metabolic and cardiovascular disorders such as diabetes, atherosclerosis, hypertension, and infarctions [4]–[8]. Non-alcoholic fatty liver disease (NAFLD) is a liver manifestation of metabolic syndrome and is composed a wide variety of disorders ranging from benign simple steatosis to progressive inflammation and fibrosis (non-alcoholic steatohepatitis; NASH) [9]. Recent evidence suggests that NAFLD is responsible for the development of hepatocellular carcinoma (HCC) [10]–[12], and the population of NAFLD-associated HCC patients is clearly growing [13]–[16]. In addition to such diseases associated with metabolic syndrome, many studies in humans and mice have shown a strong correlation between obesity and autoimmune diseases, which are accompanied by increased levels of autoantibodies [17]–[22]. In addition, it is known that obesity decreases multiple types of immune function including natural killer (NK) cell cytotoxicity, maturation of macrophages, and polymorphonuclear bactericidal capacity, resulting in increase in susceptibility to bacterial and viral infection [23]. Therefore, the development of therapeutic strategies to regulate obesity is strongly desired.

Recently, we found that apoptosis inhibitor of macrophage (AIM), a circulating protein initially identified as a supporter of macrophage survival [24], plays a preventive role in obesity progression [25]. AIM is produced solely by tissue macrophages under transcriptional regulation by nuclear receptor liver X receptor/retinoid X receptor (LXR/RXR) heterodimers [24], [26]–[28]. As a secreted molecule, AIM is detected in human and mouse blood at varying levels [24], [25], [29]–[34]; and circulating AIM increases with the progression of obesity in mice fed a high fat diet (HFD) [25]. Circulating AIM is incorporated into adipocytes via CD36-mediated endocytosis, where it directly binds and inactivates cytoplasmic fatty acid synthase (FASN) [25]. This response reduces the production of lipid droplet-coating proteins, such as fat-specific protein 27 (FSP27) and perilipin, thereby decreasing triacylglycerol (TG) deposition within adipocytes [25], [35], [36]. Accordingly, adipocyte hypertrophy was more advanced and the mass of visceral adipose tissues was greater in *AIM* ^−/−^ mice than in *AIM^+/+^* mice fed an HFD. This HFD-induced hyperobese phenotype was abolished by administering recombinant AIM (rAIM) to the *AIM* ^−/−^ mice [25], [35], [36]. These findings suggest that AIM might be an effective therapeutic tool to regulate obesity progression.

Interestingly, AIM associates with the immunoglobulin (Ig)M pentamer in blood, an association which protects AIM from renal excretion and thus maintains relatively high circulating concentrations (∼2.5−10 µg/mL) [22], [37]. Consistently, a strong correlation between AIM and natural IgM levels in the blood has been found in both humans and mice [22], clearly indicating that levels of circulating AIM are dependent on natural IgM levels, except in cases in which *AIM* expression is abrogated in macrophages. Accordingly, administration of AIM into mice lacking circulating IgM, such as *recombinase-activating gene 1*-deficient (*RAG1* ^−/−^) mice or secreted-type IgM-deficient (Δsµ) mice [38], was not effective for sustained increases in AIM levels, as the free AIM was rapidly excreted in the urine [22]. Similarly, increases in blood AIM levels in wild-type animals, which have normal levels of IgM, may not be achieved by AIM administration, as most circulating IgM is already associated with endogenous AIM [22].

In this study, we evaluated new strategies for increasing circulating AIM levels in the presence or absence of IgM. We synthesized an IgM-Fc protein as well as a binding complex of Fc and AIM and evaluated their ability to augment circulating AIM levels without any undesired immune activation. In view of the potential for future use in humans, we used human IgM-Fc and human AIM for this study in a mouse model.

## Materials and Methods

### Mice

*AIM* ^−*/*−^ mice [24] had been backcrossed to C57BL/6 (B6) for 15 generations before used for experiments. It is noteworthy that no genetic locus responsible to obesity has been found near the AIM gene. Δsµ mice were purchased from The Jackson Laboratory. All mice were maintained under an SPF condition. All animal experiments were carried out in strict accordance with the recommendations in the Guide for the Care and Use of Laboratory Animals of the National Institutes of Health. The protocol was approved by the Committee on the Ethics of Animal Experiments of the University of Tokyo (Permit Number: P10–143). All surgery was performed under sodium pentobarbital anesthesia, and all efforts were made to minimize suffering.

### Antibodies

Antibodies used in this study are as follows: AIM (Rab2; rabbit polyclonal anti sera); FLAG (M2, SIGMA, St. Louis, MO); c-Myc (QED Bioscience, San Diego, CA); HA (clone 3E10, Roche, Basel, Switzerland); F4/80 (Clone BM8, Invitrogen, Carlsbad, CA); Hoechst 33342 (Invitrogen); mouse IL-6 (clone MP5-20F3, R&D System), mouse C1q (rabbit polyclonal antibody, Abcam); α1-antitrypsin (chicken polyclonal antibody, Abcam). Secondary antibodies: Cy3 goat anti-rat IgG antibody (Chemicon, Billerica, MA); Alexa Fluo 488 chicken anti-rabbit IgG antibody (Molecular Probes, Eugene, OR). For ELISA, antibodies to measure human IgM, mouse IgM, and mouse IgG were purchased from BETHYL laboratories (Montgomery, TX).

### Biacore Analysis

The interaction of human IgM-Fc monomer with immobilized human AIM was examined at 25°C using a Biacore 3000 (GE Healthcare, Little Chalfont, UK) surface plasmon resonance instrument. Human recombinant (r)AIM was covalently immobilized at pH 4.0 to 2800 resonance units (RU) in one flow cell on a CM5 sensor chip (GE Healthcare), whereas the control flow cell underwent no treatment. The carboxymethyl groups of dextran were activated using 1-ethyl-3-(3-dimethylaminopropyl)-carbodiimide hydrochloride and N-hydroxysuccinimide. Remaining reactive sites were blocked by ethanolamine, and the sensor chip was washed with 100 mM borate pH 8.5, 500 mM NaCl to remove non-covalently bound ligand. The IgM-Fc monomer was diluted in a running buffer (10 mM HEPES pH 7.4, 150 mM NaCl, 3 mM EDTA, 0.05% Tween-20) at the concentration ranged from 40 µM to 1280 µM. Resulting IgM-Fc was injected to the rAIM-immobilized- and control flow cells at a flow rate of 20 µL/min for 2.5 min. After the injection, the IgM-Fc was allowed to dissociate in the running buffer for 3 min, and then the capture surface was regenerated with 20 mM Glycine-HCl pH 2.5, 1 M NaCl, 0.1% Tween-20 for 1 min. Results were analyzed using BIAevaluation software (version 4.1). The differences in binding responses on the rAIM-immobilized flow cell and the control flow cell were fit to the 1∶1 Langmuir binding model to determine kinetic and affinity constants.

### Generation and Purification of Synthesized and Recombinant Proteins

For FL-hFc, HEK 293T cells were co-transfected with pCAGGS-FL-hFc plasmid and pCAGGS-hIgJ-Myc plasmid, and cultured in DMEM+GlutaMax-I (Gibco, Carlsbad, CA) supplemented with 10% FBS for 3 days. FL-hFc was purified from culture supernatant using an anti-FLAG antibody (M1; clone 4E11, SIGMA) conjugated column. The purification column was prepared by coupling the HiTrap NHS-activated HP column (1 ml agarose volume; GE-Healthcare) with 10 mg of antibody. The column was equilibrated with TBS/Ca buffer (50 mM Tris pH 7.4, 150 mM NaCl, 10 mM CaCl_2_), and the calcium concentration of the sample was adjusted by adding 1/9 volume of 10x TBS/Ca buffer. After loading the sample, the column was washed with TBS/Ca buffer containing 0.05% Tween-20. Captured protein was eluted with Glycine-HCl pH 3.0, and neutralized with 1 M Tris-HCl pH 8.5. Purified protein was concentrated using Amicon Ultra 100k (Millipore, Billerica, MA).

For hFc-AIM, HEK 293T cells were separately transfected with pCAGGS-hAIM-HA plasmid, or with pCAGGS-FL-hFc plasmid and pCAGGS-hIgJ-Myc plasmid. After transfection, these cells were co-cultured for 3 days. The hFc-AIM was sequentially purified by two types of columns. The culture supernatant was first passed over the HiTrap NHS-activated HP column containing an anti-hAIM monoclonal antibody (clone #7, Transgenic Inc., Tokyo). The column was equilibrated with 20 mM Tris pH 7.5, 0.1 M NaCl, 0.1 mM EDTA. After loading the supernatant, the column was washed with the equilibration buffer containing 0.05% Tween-20. Captured protein was eluted with Glycine-HCl pH 3.0, and neutralized with 1 M Tris-HCl pH 8.5. The overall elutants were then passed over the column containing an anti-FLAG antibody to remove free hAIM.

For monomeric human IgM-Fc, HEK 293T cells were transfected with pSecTag2-Fc (CH2-CH3-CH4 domain) -Myc plasmid. This construct was designed to express secretion form of human IgM-Fc (CH2-CH4) with a Myc tag at the C terminus. The monomeric IgM-Fc was purified from culture supernatant using an anti-c-Myc antibody conjugated agarose column (SIGMA). After loading the supernatant, the column was washed with PBS containing 0.05% Tween-20, and the captured monomeric IgM-Fc was eluted with 0.1 M ammonium hydroxide pH 11.3, and neutralized with 1 N acetic acid. The elutants were concentrated and the resolving buffer was exchanged to PBS using Amicon Ultra 10k (Millipore).

### Immunoprecipitation

30 µl of mouse serum was incubated with 10 µl of anti-FLAG antibody conjugated affinity gel (SIGMA) in 200 µl of total volume at 4°C overnight. The precipitates were washed with wash buffer (1% NP-40 in PBS containing protease inhibitors) for 5 times, and resolved in 20 µl of 2xSDS sample buffer containing methanol. Samples were heated at 95°C for 5 minutes, and loaded on SDS-PAGE for immunoblotting.

### Histology

The anesthetized mice were perfused with 4% paraformaldehyde (PFA) in PBS, and the epididymal white adipose tissue was post-fixed in 4% PFA/PBS at 4°C overnight. The tissues were then treated in 30% sucorese at 4°C overnight. The specimens were embedded in Tissue-TEK O.C.T. compound (Sakura, Torrance, CA), and 14 µm sections were made using a cryostat microtome (CM3050S; LEICA, Wetzlar, Germany). Histological analysis was performed using a confocal microscope (FV10i; Olympus, Tokyo).

## Results

### AIM Bound to IgM-Fc

**Figure 1:**
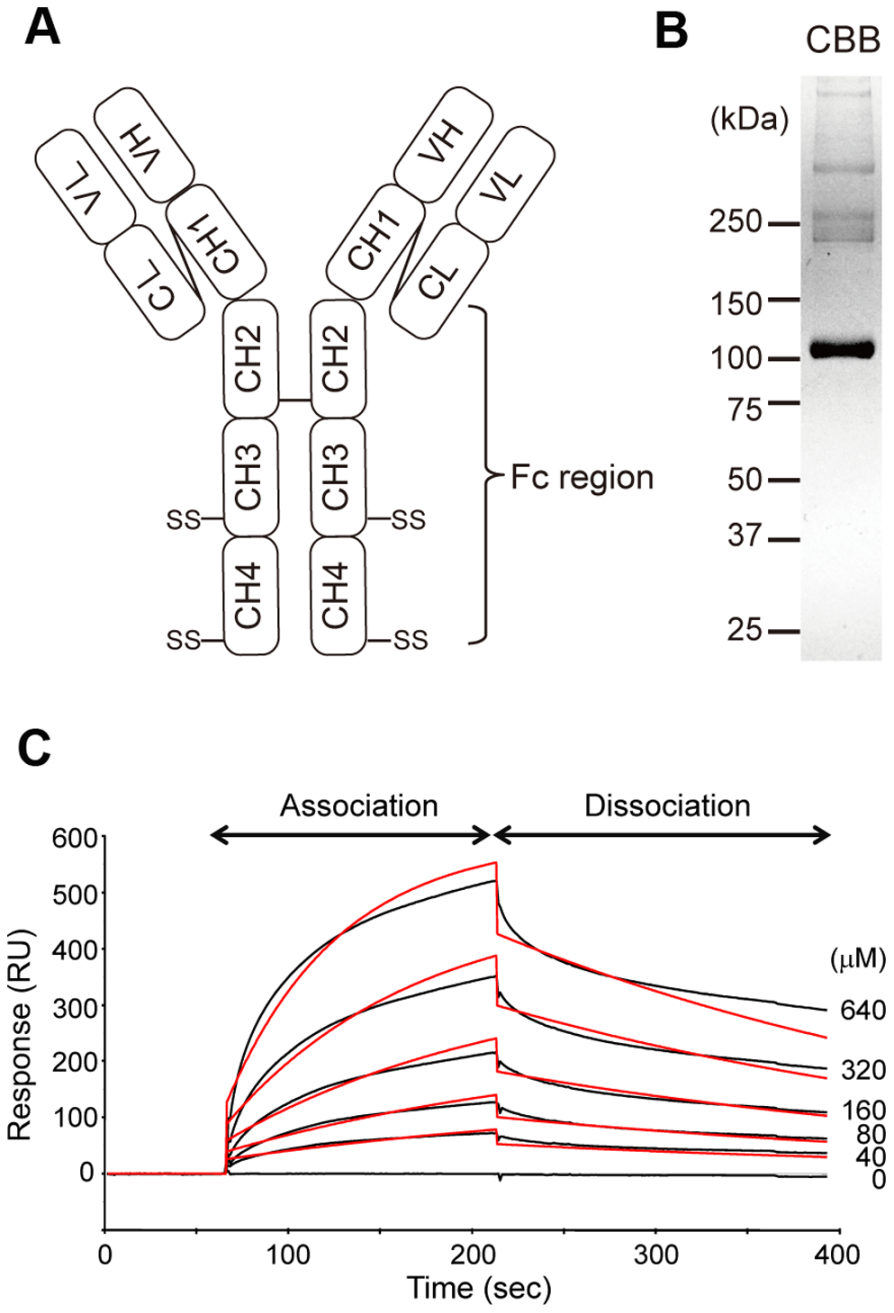
Kinetic analysis of IgM-Fc binding to AIM. (A) Diagram illustrating the relative positions of the domains of an IgM molecule. (B) We employed monomeric IgM-Fc (110 kDa) for the Biacore experiments. (C) Monomeric IgM-Fc (0, 40, 80, 160, 320, and 640 µM) was injected into flow cells, and the flow cells were washed with a running buffer to monitor dissociation (black). Binding responses were fit to the 1∶1 Langmuir binding model to determine kinetic and affinity constants (red).

In a previous report, we suggested that AIM may bind to the Fc portion of IgM, as AIM associates with different monoclonal IgM clones regardless of the type of the variable region. To determine whether AIM harbours a significant binding region recognizing IgM-Fc, here we employed the Biacore system. As schematized in Fig. 1A, the free constant (Fc) region of the IgM heavy chain consists of three out of four conserved domains (CH1-CH4); CH1 is associated with the IgM light chain through an S-S boundary, and the CH2-CH4 region is free of the light chain. Like other types of immunoglobulin, two sets of the heavy-light chains form a dimer linked at the cysteine within CH2. Under physiologic conditions, IgM forms a pentamer involving two S-S boundaries in CH3 and CH4 (Fig. 1A). Because the pentameric form of IgM is not suitable for the Biacore analysis, we generated a monomeric IgM by adding a Myc Tag at the C-terminus (hFc-Myc; ∼100 kDa), which interfered with the formation of the pentamer (Fig. 1B).

Human rAIM was immobilized on a sensor chip surface, and varying concentrations of the hFc-Myc were injected across the surface. Changes in the index of refraction at the surface where the binding interaction occurs were detected and recorded as resonance units (RU). As shown in Fig. 1C, curves were generated from the RU trace and were evaluated by fitting algorithms that compare the raw data to well-defined binding models. These fits allowed determination of the apparent affinity of the binding between AIM and the synthesized IgM-Fc. The resulting association rate constant (Ka) was 2.05×10^4^ (/molar second: Ms), the dissociation rate constant (Kd) was 3.15×10^−3^ (/s), and the dissociation-association rate constant (K_D_) was 1.53×10^−7^ (M).

### Creation of a Human IgM-Fc Pentamer and its Association with AIM

**Figure 2:**
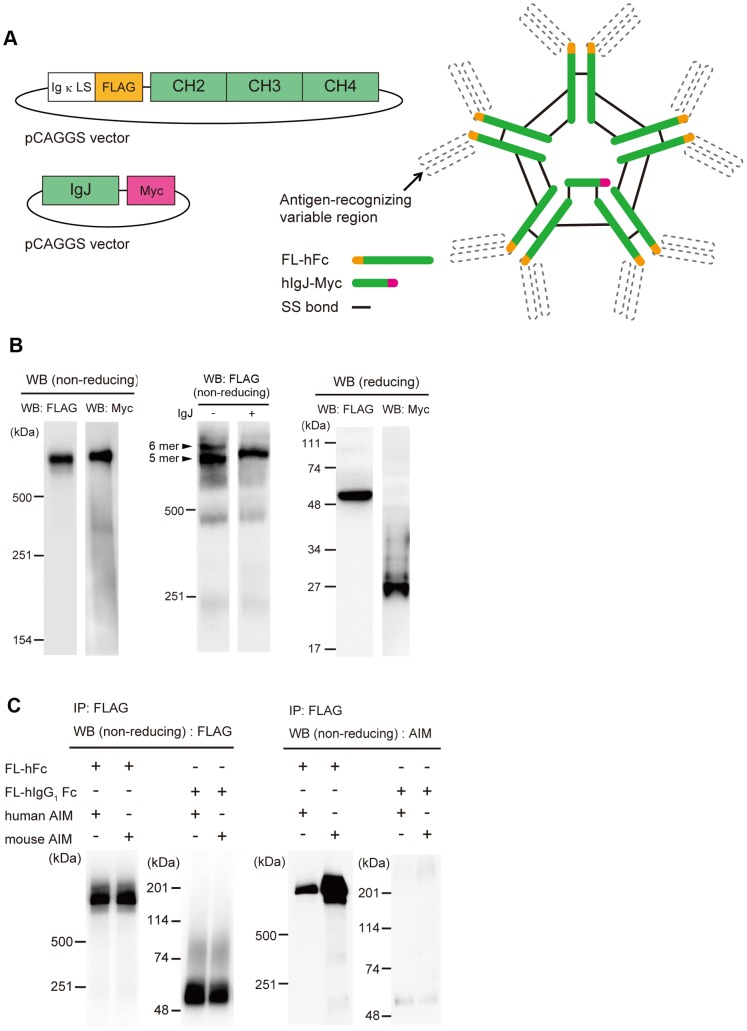
The human IgM-Fc pentamer can associate with both human and mouse AIM. (A) IgM is secreted as a pentamer, and each pentamer contains one IgJ chain. We created an IgM-Fc pentamer lacking the antigen-recognition regions. (B) *Left*: Non-reducing western blotting (WB) showed that the FL-hFc formed a pentamer (600 kDa) and that the pentamer was accompanied by an IgJ chain. *Middle*: Non-reducing WB of culture supernatant from HEK293T cells transfected with only pCAGGS-FL-hFc plasmid. Without IgJ chain, the FL-hFc formed a pentamer and hexamer (indicated by arrows). *Right*: When sodium dodecyl sulphate-polyacrylamide gel electrophoresis (SDS-PAGE) was performed under reducing conditions, signals at the sizes expected for a single FL-hFc chain (55 kDa) and IgJ-Myc (25 kDa) were detected. (C) The association of human IgM-Fc pentamer and AIM was examined by a coimmunoprecipitation (IP) assay with anti-FLAG antibody. Precipitates were analysed by non-reducing WB. Human IgG_1_ Fc was used as a negative control.

Having confirmed that AIM bound to Fc with sufficient affinity, we then created recombinant human IgM-Fc in a pentameric form and investigated its association with AIM. We fused a FLAG tag to the N-terminus of the CH2 domain (FL-hFc, Fig. 2A). This design allowed not only formation of the pentamer of the IgM, but also efficient association with the IgJ chain. Interestingly, the IgM pentamer lacking IgJ does not associate with AIM. Both FL-hFc and Myc-tagged IgJ expression vectors (Fig. 2A) were co-transfected into HEK293T cells, and the culture supernatant was assessed by immunoblotting. When tested for FL-hFc under nonreducing conditions with an anti-FLAG antibody, the signal was detected at a high molecular weight (∼ 600 kDa), suggesting that the FL-hFc formed a pentamer (Fig. 2B). This pentamer was accompanied by the IgJ chain, as immuneoblotting with an anti-Myc antibody produced a signal at an identical position (Fig. 2B). Although expression of FL-hFc without IgJ resulted in formation of both pentamer and hexamer, the pentamer formation was predominant when IgJ was co-expressed (Fig. 2B). When assessed under reducing conditions, signals at the expected sizes for the single FL-hFc chain or IgJ-Myc were observed with the anti-FLAG or anti-Myc antibodies, respectively (Fig. 2B). Thus, we successfully created the human IgM-Fc pentamer combined with IgJ.

To assess the binding of AIM to the synthesized IgM-Fc pentamer, the culture supernatant from HKT293T cells expressing both FL-hFc and IgJ-Myc was incubated with purified human or mouse rAIM, and immune-precipitation of FL-hFc was performed with an anti-FLAG antibody. As shown in Fig. 2C, both human and mouse AIM co-precipitated with the Fc pentamer, as assessed with a polyclonal anti-AIM antibody (Rab2). Thus, the synthesized IgM-Fc pentamer formed a complex with AIM similar to that with the intact IgM pentamer. Human IgG_1_-Fc did not bind to AIM (Fig. 2C), in accordance with our previous observation showing no apparent association of AIM with IgG in the blood.

### Restoration of Circulating AIM by Synthesized IgM-Fc in the Absence of Blood IgM

**Figure 3:**
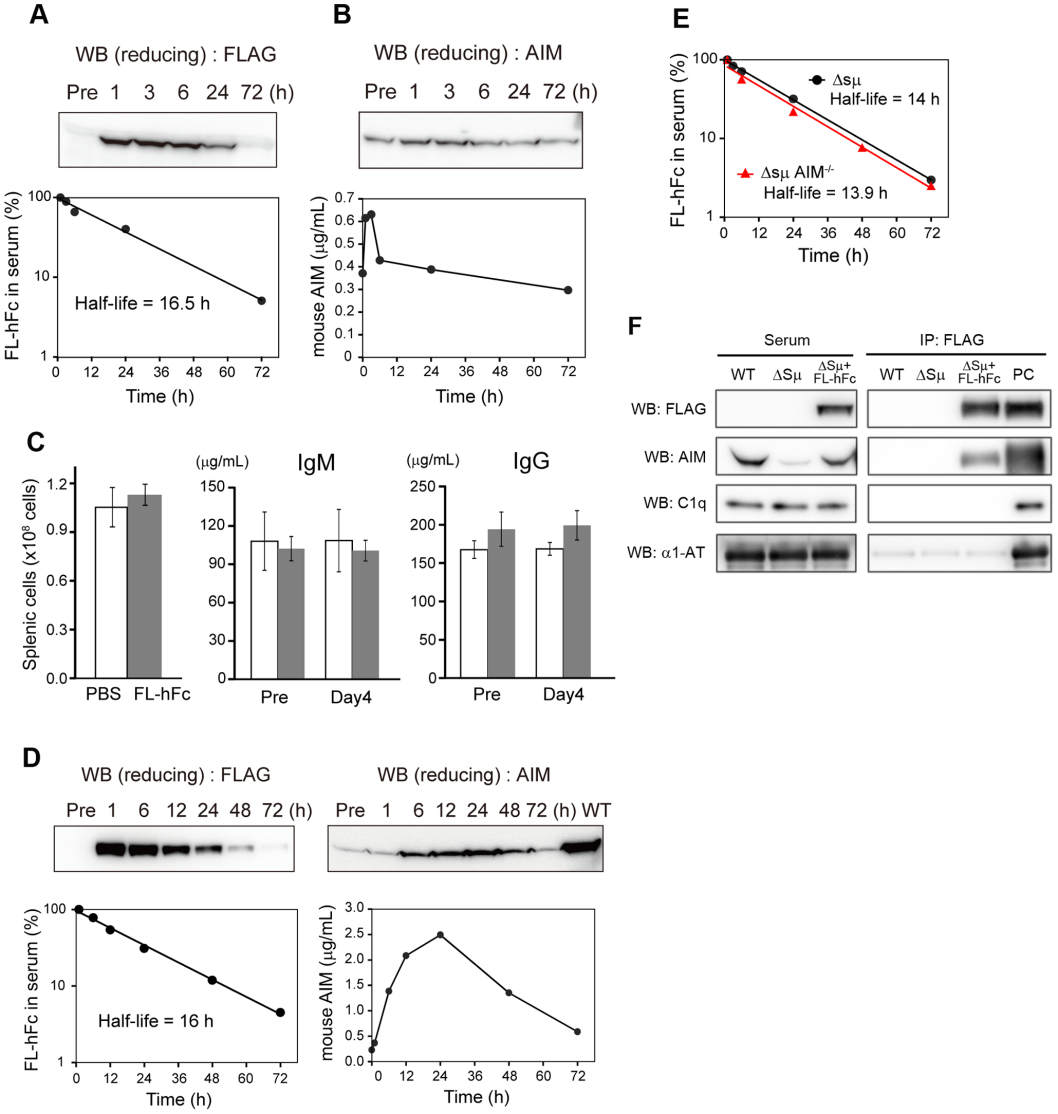
Augmentation of blood AIM levels in IgM-deficient mice by treatment with synthesized IgM-Fc. Representative curves of FL-hFC levels (A) or mouse AIM levels (B) in serum of Δsμ mice injected intravenously with 100 µg of purified FL-hFc. (C) There were no differences in the absolute number of splenic cells or serum IgM and IgG levels at 4 days after administration of FL-hFc (100 µg) or PBS to C57BL/6 (B6) mice (*n* = 3 per group). (D) Representative curves of FL-hFC levels or mouse AIM levels in sera of Δsμ mice injected intravenously with 1 mg of purified FL-hFc. Twenty-four hours after injection, the circulating AIM level increased to 35% of that in wild-type (B6) mice. (E) Representative curves of FL-hFC levels in sera of Δsμ mice or Δsμ *AIM* ^−*/*−^ mice injected intravenously with purified FL-hFc. Black circles and red triangles represent the serum FL-hFC levels of the Δsμ mice and the Δsμ *AIM* ^−*/*−^ mice, respectively. (F) FL-hFc was immnoprecipitated using an anti-FLAG antibody from serum of Δsμ mice injected intravenously with 100 µg of purified FL-hFc (Δsμ+FL-hFc), and the precipitates were tested for AIM, C1q, and α1-antitrypsin (α1-AT) by Western blotting (WB). Sera of Δsμ mice without injection of FL-hFc (Δsμ) and wild-type (WT) mice was used as a control. Results for WB of serum are also presented (left panel). PC: positive control for WB; FL-hFc (for FLAG), rAIM (for AIM), WT mouse serum (for C1q and α1-AT), respectively.

We previously demonstrated that complex formation with IgM pentamer prevents the excretion of AIM into urine, resulting in stabilization of blood AIM. Accordingly, the circulating AIM level was far lower in mice lacking natural IgM, such as secreted-type IgM deficient (Δsμ) mice, than in wild-type mice, despite comparable levels of AIM production by tissue macrophages in both types of mice. We, therefore, addressed whether the synthesized IgM-Fc may restore AIM levels in the absence of IgM. We injected 100 µg of purified FL-hFc intravenously into Δsμ mice and determined AIM levels in the blood by immunoblotting. Note that our FL-hFc did bind to mouse AIM (Fig. 2C). As shown in Fig. 3A, the half-life of the injected FL-hFc was 12–16 h, which is quite comparable to that of natural IgM. In parallel, the AIM level continued to increase until 24 h after FL-hFc administration (Fig. 3B). At 72 h after injection, the AIM level was still higher than the basal level. Thus, the synthesized IgM-Fc stabilized AIM in blood, resulting in augmentation of circulating AIM levels. The administration of FL-hFc did not cause undesired immune activation, as none of the immune parameters, such as absolute number of splenic cells and serum Ig levels, changed in response to FL-hFc (Fig. 3C).

We also challenged the Δsμ mice with a larger amount (1 mg) of FL-hFc and obtained a similar result with an identical half-life of the injected Fc protein (Fig. 3D). At 24 h after the injection, the circulating AIM level increased to 35% of that in the wild-type mouse (Fig. 3D). Again, no undesired immune activation was induced by the injection (data not shown). When FL-hFc was injected into mice doubly deficient for secreted type IgM and AIM (Δsμ *AIM* ^−*/*−^ mice), the half-life of injected FL-hFc was comparable to that in Δsμ mice (Fig. 3E). This result suggests that association with AIM did not influence to the stability of IgM-Fc in the blood.

In blood, AIM is a participant of IgM-immune complex containing many molecules including complementary factor 1q (C1q) and α1-antitrypsin. To assess whether the FL-hFc also formed a complex composed by such molecules, we immunoprecipitated the FL-hFc from the Δsμ mouse serum injected with FL-hFc using an anti-FLAG antibody, and the precipitates were tested for AIM, C1q, and α1-antitrypsin. Interestingly, either C1q or α1-antitrypsin was not associated with the complex of FL-hFc and endogenous AIM (Fig. 3F).

### Efficient Augmentation of Circulating AIM by Administration of Fc-AIM Complex

Having observed that synthesized IgM-Fc administration rescued the circulating AIM from an unstable state caused by low IgM levels, we then wondered whether it was possible to augment AIM levels in the presence of normal IgM levels. In wild-type mice, because most circulating IgM pentamers are occupied by endogenous AIM and lack sufficient “space” for additional AIM, any administrated AIM is rapidly excreted into urine and produces no steady increase in circulating AIM levels.

**Figure 4:**
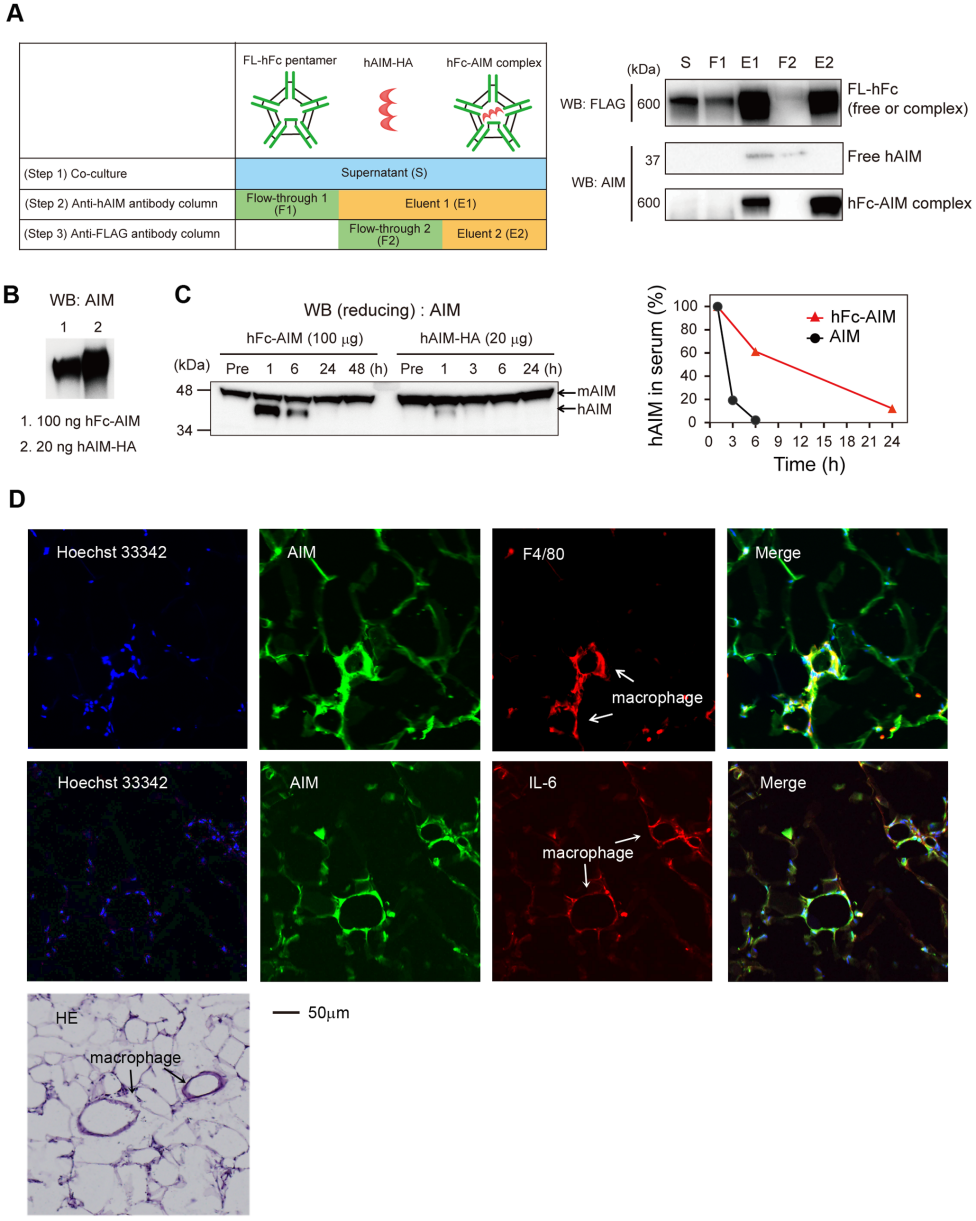
The Fc-AIM complex serves to prolong the half-life of AIM. (A) The strategy for purification of the hFc-AIM complex. S: culture supernatant; F1 and E1: flow-through and eluent from the anti-hAIM monoclonal antibody column; F2 and E2: flow-through and eluent from the anti-FLAG (M1) antibody column. (B) 100 µg of hFc-AIM contains about 20 µg of hAIM. (C) 100 µg of purified hFc-AIM complex or 20 µg of purified hAIM was intravenously injected into wild-type (B6) mice, and the half-lives of the injected hAIM were compared by immunoblotting. *Left*: Injected hAIM (37 kDa) can be distinguished from endogenous mouse AIM (45 kDa) by molecular weight. *Right*: The values of hAIM were normalized so that the values at 1 h after injection corresponded to 100%. Red triangles and black circles represent the serum hAIM levels of the mice injected with hFc-AIM and hAIM alone, respectively. (D) Specimens of epididymal white adipose tissue from the obese *AIM* ^−*/*−^ mouse administered 500 µg of hFc-AIM were stained for AIM (green) and F4/80 (red), and counterstained with Hoechst 33342 (blue). The crown-like structures formed by recruited macrophages are indicated by arrows. Different sections were stained for AIM (green) and IL-6 (red), as well as with HE.

We therefore generated a complex comprising FL-hFc pentamer and human AIM (hFc-AIM). HEK293T cells expressing either FL-hFc and IgJ-Myc or HA-tagged human AIM (hAIM) were co-cultured, and the culture supernatant was passed through two types of columns containing an anti-hAIM monoclonal antibody or an anti-FLAG antibody to specifically purify the hFc-AIM complex (Fig. 4A). At each step during the purification procedure, we confirmed the elimination of uncoupled FL-hFc or hAIM by immunoblotting with anti-HA (for AIM) and anti-FLAG (for FL-hFc) antibodies (Fig. 4A).

Purified hFc-AIM (100 µg) was intravenously injected into wild-type C57Bl/6 mice, and circulating hAIM levels were analyzed over time by immunoblotting with a polyclonal antibody (Rab2) that reacts to both mouse and human AIM. Mouse and human AIM can be distinguished by size (molecular weights, 45 kDa and 37 kDa, respectively) mainly because of the difference in the degree of glycosylation. As a control, we also injected hAIM alone into different wild-type mice to compare the half-life of the administrated hAIM in the blood. Note that we injected 20 µg of hAIM, which corresponds to the amount of hAIM contained in 100 µg of hFc-AIM (Fig. 4B). As shown in Fig. 4C, when hAIM alone was injected, its half-life was less than 2 h in mouse blood, which was similar to that observed previously. In contrast, when hFc-AIM was injected, the half-life of hAIM was 12 h, or six times longer than that observed when hAIM alone was injected (Fig. 4C). Hence, the preservation of hAIM by the Fc-AIM complex in the blood was dependent on the stability of Fc, as the half-life of hAIM from hFc-AIM was similar to that with FL-Fc. Thus, circulating AIM levels could be increased by employing the Fc-AIM complex.

### AIM Administered as an Fc-AIM Complex Behaved Similarly to Endogenous AIM

Lastly, we investigated whether hAIM provided as hFc-AIM functions as endogenous AIM does. To this end, we assessed incorporation of administrated hAIM into adipocytes in visceral fat tissue. We injected 500 µg of hFc-AIM intravenously into obese *AIM* ^−*/*−^ mice and analyzed epididymal fat tissues histologically for hAIM incorporation. As presented in Fig. 4D, the adipocytes stained positively for AIM. Consistent with the fact that AIM is also incorporated into macrophages, adipose tissue macrophages also stained positively for AIM (Fig. 4D). Infiltrating M1 macrophages that expressed IL-6 also incorporated AIM (Fig. 4D). These results suggest that AIM administrated as an hFc-AIM complex may function effectively in recipients.

## Discussion

Our findings suggest a new strategy for increasing circulating AIM levels that may serve as a basis for therapy for certain disorders caused by low AIM. Firstly, synthesized IgM-Fc forms a pentamer containing IgJ and associates with AIM. A previous study demonstrated that the maximum Ka for the binding of a protein antigen by its antibody was approximately 10^5^–10^6^ and the Kd of the naturally prepared antibody molecule was fixed at 10^−3^–10^−2^ [39]. Hence, the AIM-Fc binding was comparable to that of an antibody-antigen interaction at relatively low affinity. Secondly, administration of synthesized Fc stabilizes and augments endogenous blood AIM in mice lacking natural IgM. In addition, this treatment does not provoke any undesired immune response. Thirdly, the Fc-AIM complex incrementally increases the circulating AIM, which appears to function similarly to endogenous AIM. These findings support the applicability of synthesized Fc and the Fc-AIM complex for therapeutically increasing AIM levels under conditions of both low and normal levels of IgM. Note that in both cases, supplementation with AIM itself did not effectively increase AIM levels because AIM uncoupled from IgM is rapidly excreted into the urine (Fig. 4C and ref. 22).

Based on the strong correlation in the blood levels of AIM and IgM [22], patients with genetic immunoglobulin deficiencies (X- or autosome-linked infantile agammaglobulinemia) or severe combined immunodeficiency (SCID), who exhibit null or very low natural IgM levels, may also show low AIM levels. Although significant association of hypoglobulinemia with obesity has not been reported, it is possible that these patients may be susceptible to progression of obesity, and thus, synthesized IgM-Fc and/or the Fc-AIM complex might be applicable to control obesity in these patients. Certainly, circulating AIM levels need first to be analyzed in these patients.

This Fc treatment may be safe, because administration of synthesized Fc did not stimulate an immune response (Fig. 3C), in contrast to whole IgM, which is likely to activate immunity because of its ability to recognize antigens. One might concern about a possibility of a late immune response caused by the synthesized Fc, as we did not investigate the immune response after more than 4 day-period post injection. This issue certainly needs to be further addressed.

On the other hand, the Fc-AIM complex may be useful in the more common situation of healthy individuals who gain weight as a result of their diet. These individuals have normal levels of natural IgM that are mostly occupied with endogenous AIM. Our results demonstrate that the Fc-AIM complex can incrementally augment circulating AIM levels. In addition, AIM provided in the form of an Fc-AIM complex was efficiently incorporated into adipose tissue, as is required to produce an anti-obese effect similar to that of endogenous AIM.

It could be argued that augmentation of circulating AIM might be associated with an increased risk of inducing chronic inflammation in fat tissue, leading ultimately to insulin resistance. Indeed, we previously reported that in mice, augmented blood AIM level along with progression of obesity (5∼7 times higher compared to that in lean mice) induces a high level of lipolysis and efflux of large amounts of fatty acids from adipocytes that stimulate toll-like receptor 4 (TLR4) expressed on the adipocytes [36]. This response induces proinflammatory adipokines, including monocyte-chemoattracting MCP-1, resulting in recruitment of inflammatory macrophages in adipose tissue [35], [36]. Excessive augmentation of AIM levels might certainly increase the risk of triggering such pro-inflammatory processes. Hence, it is important to frequently measure AIM levels during the administration of synthesized Fc or Fc-AIM and to regulate them so as not to far exceed the normal range (2.5–10 µg/mL in serum) [22].

Lastly, the half-life of augmented AIM was about 16 h, which is sufficiently long for a synthesized Fc or Fc-AIM complex to be considered for therapeutic application. The preservation of AIM is dependent on the stability of Fc in blood; therefore, identification of strategies to regulate the metabolism of Fc would help prolong the half-life of AIM. Further studies to overcome this issue are required.
